# Novel Molecular and Computational Methods Improve the Accuracy of Insertion Site Analysis in Sleeping Beauty-Induced Tumors

**DOI:** 10.1371/journal.pone.0024668

**Published:** 2011-09-13

**Authors:** Benjamin T. Brett, Katherine E. Berquam-Vrieze, Kishore Nannapaneni, Jian Huang, Todd E. Scheetz, Adam J. Dupuy

**Affiliations:** 1 Center for Bioinformatics and Computational Biology, Roy J. and Lucille A. Carver College of Medicine, University of Iowa, Iowa City, Iowa, United States of America; 2 Department of Anatomy and Cell Biology, Roy J. and Lucille A. Carver College of Medicine, University of Iowa, Iowa City, Iowa, United States of America; 3 Department of Biomedical Engineering, Roy J. and Lucille A. Carver College of Medicine, University of Iowa, Iowa City, Iowa, United States of America; 4 Department of Statistics and Actuarial Science, Roy J. and Lucille A. Carver College of Medicine, University of Iowa, Iowa City, Iowa, United States of America; 5 Department of Biostatistics, Roy J. and Lucille A. Carver College of Medicine, University of Iowa, Iowa City, Iowa, United States of America; 6 Department of Ophthalmology and Visual Sciences, Roy J. and Lucille A. Carver College of Medicine, University of Iowa, Iowa City, Iowa, United States of America; 7 Department of Pathology, Roy J. and Lucille A. Carver College of Medicine, University of Iowa, Iowa City, Iowa, United States of America; Southern Illinois University School of Medicine, United States of America

## Abstract

The recent development of the Sleeping Beauty (SB) system has led to the development of novel mouse models of cancer. Unlike spontaneous models, SB causes cancer through the action of mutagenic transposons that are mobilized in the genomes of somatic cells to induce mutations in cancer genes. While previous methods have successfully identified many transposon-tagged mutations in SB-induced tumors, limitations in DNA sequencing technology have prevented a comprehensive analysis of large tumor cohorts. Here we describe a novel method for producing genetic profiles of SB-induced tumors using Illumina sequencing. This method has dramatically increased the number of transposon-induced mutations identified in each tumor sample to reveal a level of genetic complexity much greater than previously appreciated. In addition, Illumina sequencing has allowed us to more precisely determine the depth of sequencing required to obtain a reproducible signature of transposon-induced mutations within tumor samples. The use of Illumina sequencing to characterize SB-induced tumors should significantly reduce sampling error that undoubtedly occurs using previous sequencing methods. As a consequence, the improved accuracy and precision provided by this method will allow candidate cancer genes to be identified with greater confidence. Overall, this method will facilitate ongoing efforts to decipher the genetic complexity of the human cancer genome by providing more accurate comparative information from Sleeping Beauty models of cancer.

## Introduction

Recent work has indicated that the human cancer genome is complex, consisting of many somatically acquired genetic and epigenetic changes [1]. A key challenge faced by the cancer genetics community is deciphering the role that this complexity plays in the etiology of human cancer. Unfortunately, we still have a limited ability to specifically identify those mutations that drive cancer initiation and progression among the larger number of passenger mutations found in an individual tumor. A subsequent goal would then be to determine how individual driver mutations cooperate to generate and maintain a tumor. Armed with this knowledge, it is thought that more effective cancer therapies can be generated to specifically target tumors.

Mouse models of cancer have become a useful tool in modeling the genetics of human cancer, allowing the investigator to test the role of mutant forms of specific candidate genes *in vivo*. Moreover, insertional mutagenesis models of cancer have shown great promise not only in the identification of novel candidate cancer genes, but also in providing insight into how specific combinations of gene mutations produce cancer [2]. Retroviral, and more recently, transposon mutagenesis models have been described that model a wide variety of tumor types in the mouse [2], [3]. The great advantage of these models is that the driver mutations are tagged by proviral or transposon sequences that facilitate their rapid identification.

Advances in DNA sequencing technology have greatly facilitated the characterization of mouse tumors induced by insertional mutagenesis. Several independent methods have been produced that utilize a ligation-mediated PCR approach to amplify proviral or transposon junction fragments from the tumor genome [4], [5]. Incorporation of barcodes in the PCR allows the products from independent samples to be mixed and directly sequenced [6], [7], [8]. This approach has dramatically increased the amount of data generated from insertional mutagenesis screens in mouse cancer models.

However, the bioinformatic analysis of data derived from tumors induced by insertional mutagenesis has also been complicated by the increased scale of DNA sequencing. For example, tumors that develop in existing mouse models continue to acquire retroviral or transposon integration events. Although ongoing insertional mutagenesis increases likely drives tumor progression in these models, the resulting genetic complexity also makes it difficult to accurately identify the collection of insertional mutations that were acquired during the early steps of transformation. Past analysis has assumed that early integration events present in the tumor-initiating cell will be present in all tumor cells. As a consequence, these early events will be clonally expanded to a greater extent compared to integration events acquired in tumor subclones. By extension, initiating integration events should be recurrently PCR-amplified and sequenced. While this is a reasonable assumption, it is also likely that PCR bias contributes to the frequency at which specific integration events are amplified and sequenced. Finally, recent work has shown that many hundreds of independent insertion events can be identified in an individual tumor sample [7], [8]. Given this complexity, suboptimal sequence depth is also likely to introduce sampling error and confound efforts to identify the clonally expanded integration events associated with early stages of tumor formation.

We have previously developed a variety of mouse models of cancer in which tumors are induced by Sleeping Beauty transposon mutagenesis [6], [7], [8], [9], [10], [11]. Currently, the identification of transposon-induced mutations in the tumors from these models uses pyrosequencing (Roche/454) [6], [7], [8], [9]. This method generally produces thousands of sequence reads for each sample analyzed — a significant improvement over standard Sanger sequencing [10], [11]. However, analysis of pyrosequencing data obtained from repeated sequence runs of the same tumor samples only identifies a portion of the insertion sites present in each tumor sample [12]. This suggests that deeper sequencing is required to capture the complexity of transposon integration sites found in SB-induced tumors.

Here we describe a new method to identify and analyze insertional mutations in tumors using Illumina sequencing. Using two independent sets of transposon-induced tumors, we have validated this method to determine the sequence depth required to generate a reproducible integration pattern from each sample to consistently identify common insertion sites (CISs) in SB-induced tumors. Our findings suggest that current methods used to perform this analysis likely lead to the identification of significant false-positive candidate cancer genes that could largely be addressed using the approach described here.

## Results

### Amplification and direct sequencing of transposon junction sequences

The initial method for identification of transposon insertions sites made use of the GS FLX sequencing platform (Roche). This platform was chosen because the longer read length (∼100 bp) was necessary to obtain sufficient sequence information to allow sample barcoding, verification of transposon structure, and mapping of the genomic junction sequence. However, the Illumina sequencing method has recently been improved to achieve longer sequence reads of 75 bases or greater. We set out to develop a method to prepare transposon junction amplicons for direct sequencing on the Illumina platform to take advantage of the significant increase in sequence depth provided by this approach (Methods S1).

The initial steps of the ligation-PCR method are similar to those previously published (Figure S1) [6]. However, the transposon-specific primer used in the nested PCR step was significantly redesigned. First, the primer is designed to bind to the sequences at the ends of the transposon inverted repeats (Figure S1). In addition, a six-base barcode was used in place of the original ten-base barcode used previously [6]. These changes reduce the amount of sequence needed for sample tracking and validation (32 bases), thus increasing the potential read length of the genomic junction sequence (40–45 bases). Along with these changes, the 5′ends of the transposon-specific and adaptor primers are tagged with the sequences needed to allow each PCR product to bind to the oligos that coat the surface of the Illumina flow cell. This modification allows the final PCR products to be directly sequenced on the Illumina platform, eliminating the need for additional library preparation steps. Another advantage of this approach is that it provides the ability for directional sequencing as a binding site for the standard Illumina genome sequencing primer is also incorporated into the transposon primer used in the nested PCR step.

We initially tested this approach using 62 SB-induced tumor samples using Illumina-based LM-PCR. These samples came from two previously described models of T-cell lymphoma — Vav-SB and CD4-SB, with 30 and 32 tumors respectively (Figure S2). All samples had previously been sequenced on the GS FLX sequencing platform, thus allowing direct comparison of the two approaches across many samples. The preparation was done using two technical replicates where both *Alu*I and *Nla*III were used to generate LM-PCR products from the left inverted repeat (IRL) and the right inverted repeat (IRR) (Figure S1). Each enzyme and sample combination was assigned a unique 6-base barcode during the final nested PCR step. An aliquot of the final LM-PCR products were analyzed by agarose gel electrophoresis to verify the quality of the sample (Figure S3). The remaining samples were then purified to remove unincorporated primers and nucleotides, and the sample concentration was determined using a Nanodrop spectrophotometer. Equal amounts of each sample (∼250 ng per sample) were then combined to generate a pooled sample. This sample was then sequenced in a single lane of a flow cell on a Illumina Genome Analyzer IIe.

Mapping the reads from the Illumina run posed several challenges. We have previously described a mapping and annotation pipeline that was developed to analyze sequence data produced by pyrosequencing [6]. The sequence reads are mapped using the BLAT algorithm in the existing pipeline. While this approach is robust, BLAT cannot map millions of short sequence reads in a reasonable timeframe. Therefore, we developed a new analysis pipeline that is optimized for the mapping and annotation of Illumina sequence data (Figure S4). This pipeline uses the Bowtie algorithm that was developed to map sequence data from short read platforms such as Illumina [13].

Our prior work has shown that SB-induced tumors harbor clonal transposon insertions that drive transformation. These insertion sites are present in nearly all tumor cells since they are responsible for initiating transformation. Thus they are clonally expanded along with the tumor cells. However, it is possible that each cell within the tumor mass contains a small number of transposon insertions that are unique to that cell since transposition is ongoing in all tumor cells. Due to the specificity and sensitivity of the LM-PCR process, we are able to amplify these rare transposon insertion events present in only a few cells. Thus the potential exists to identify hundreds of such background transposon insertion events in each tumor sample. Consistent with this expectation, analysis of the Vav-SB and CD4-SB Illumina sequence data showed that roughly ∼95% of mapped integration sites were represented by 25 or fewer sequence reads. By contrast, the remaining 5% of transposon integration sites contributed ∼70% of the mapped sequences — an indication that clonal expansion of specific transposon integration sites is occurring within the tumor.

Our ultimate goal is to identify candidate cancer genes that are frequently mutated by transposon insertions within SB-induced tumors. The current computational methods used to identify these candidate cancer genes generally work by identifying non-random clusters of insertion events called CISs. The identification of CISs uses statistical methods that calculate the expected frequency of insertion events within a defined genetic interval based on the total number of insertion events found throughout the genome. Therefore the inclusion of large numbers of background insertion events will dilute the insertion profile from tumor cells, thus decreasing the sensitivity and accuracy of CIS analysis. Therefore, we investigated methods to identify and remove background insertion events prior to CIS analysis.

**Figure 1:**
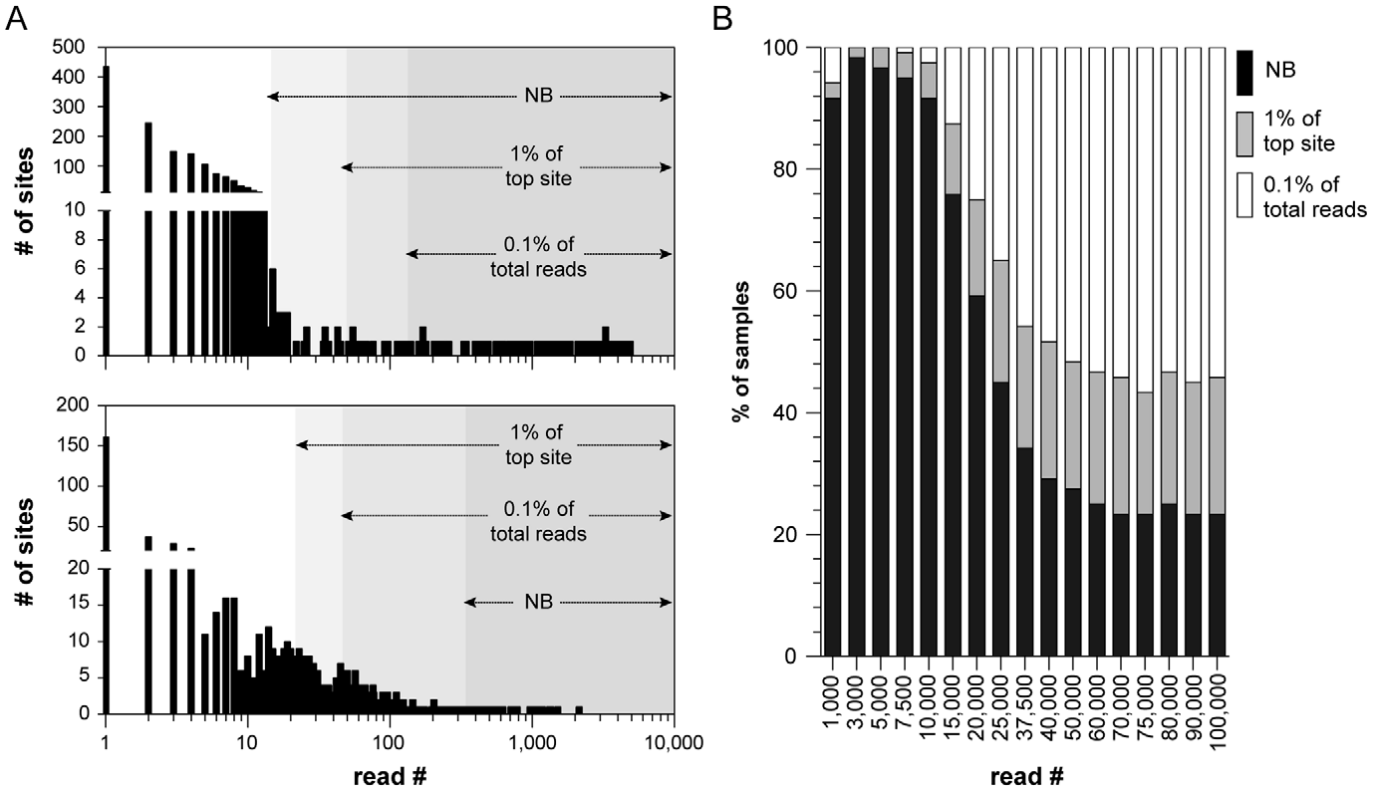
Dynamic filtering to remove background transposon insertion events. (A) Read distributions from two independent tumor samples are shown along with the calculated cutoff points using three independent methods: negative binomial (NB), 1% of the top (*i.e.* most abundant) site and 0.1% of total reads (B) An experiment to simulate a variety of sequence read depths shows that the cutoff method used by the analysis pipeline is influenced by read depth.

Initially, we examined the distribution of reads across the integration sites identified in each sample and found that the distribution of reads differed between individual samples. In addition, the distribution of reads varied between the results obtained using different restriction enzymes (*i.e. Alu*I or *Nla*III) for a single sample. Nevertheless, Illumina sequencing identified significantly more insertion events when compared directly to 454 sequencing results obtained from identical samples (Figure S5). Because of the varied read distributions, we developed three different methods to identify and remove background insertion events. Recently published ChIP-Seq algorithms use a negative binomial distribution to identify background sequences that do show enrichment [14]. Since SB data sets have a similar distribution to ChIP-Seq experiments, a negative binomial distribution (NB) was fit to all integration sites found in each barcoded sample represented by three or fewer reads. This process is then used to identify the set of integrations that are more prevalent than expected based upon the NB-estimated background (Fig. 1). These sites are defined as clonally expanded events, and all other sites are discarded.

While the NB procedure provides a reliable method to identify clonal sites, the read distribution in many samples does not adequately fit a negative binomial distribution. This is particularly true at higher read depths (Fig. 1). In these situations, the NB method fails to exclude many background insertion events. Therefore, two alternative approaches can be applied. The first method requires all clonal insertion events have a read count that is at least 1% of the most abundant insertion site. The final method requires that all clonal insertion sites be represented by a minimum of 0.1% of the total reads for the sample. As shown in Figure 1, the stringencies of these three methods vary depending on the read distribution and depth. Therefore, the analysis pipeline uses a more dynamic filtering process by calculating the read cutoff for each sample using all three methods and then applying the highest cutoff value. This dynamic cutoff method eliminates the greatest number of background insertion events, thus providing the most stringent definition of clonal transposon insertion events (Table S2).

### Determining the accuracy of Illumina-based LM-PCR

**Figure 2:**
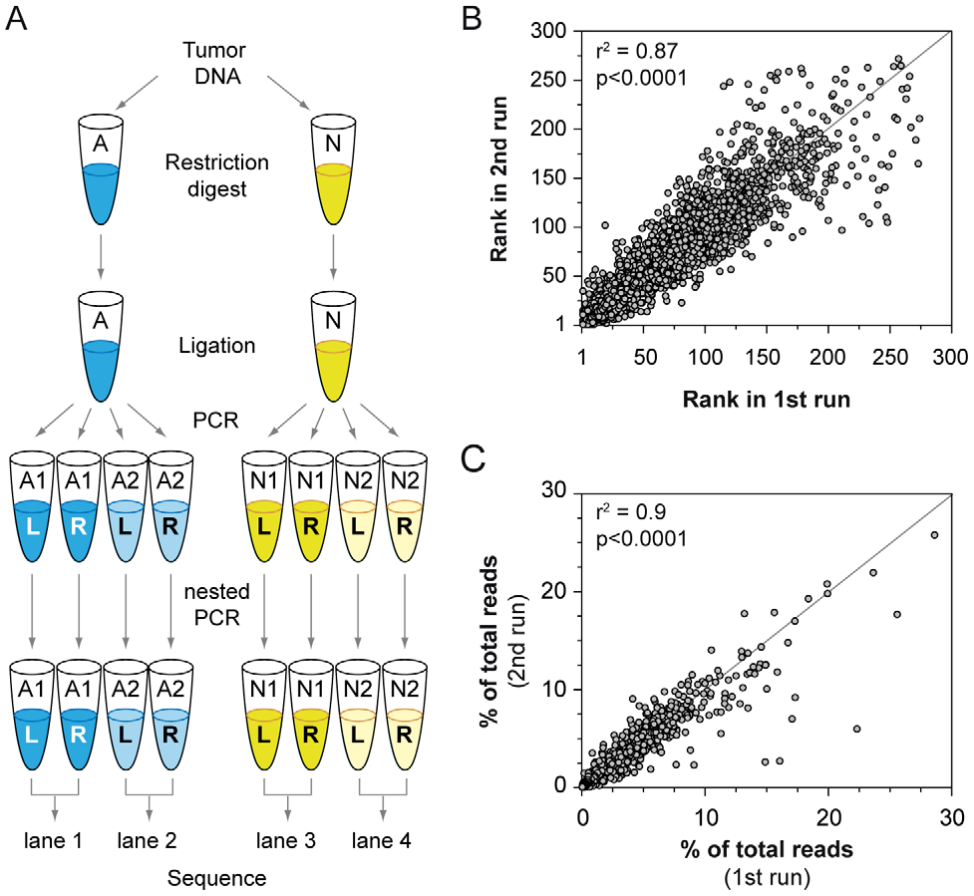
Illumina-based LM-PCR analysis consistently identifies transposon insertions in SB-induced tumors. A total of 62 T-cell lymphomas induced by SB mutagenesis using a ligation-mediated PCR approach were analyzed. Two technical replicates were performed to assess the consistency of the results (A). The results of the technical replicates were compared to assess the reproducibility of the approach. Both the rank (B) and abundance (C) of insertion sites showed a strong positive correlation between the replicate runs.

The lack of consistency seen in the 454-based LM-PCR method could be caused by variations in sample preparation or sampling error due to insufficient read depth. To test the former, we compared the results obtained in the technical replicates (Fig. 2). First, clonal transposon insertion events were identified for all samples as described previously. Next, the clonal transposon insertion sites were rank ordered based on the frequency at which each site was sequenced. The ranks from each technical replicate across all samples were compared, and a significant positive correlation (r^2^ = 0.87) was observed (Fig. 2B). Next, we calculated the percentage of total reads found for each clonal insertion site and compared these values for each site between the two replicates. Again, a strong positive correlation (r^2^ = 0.9) was observed between the technical replicates (Fig. 2C). These results suggest that variation in sample preparation does not contribute significantly to the read distribution.

### Determining optimal sequence depth

**Figure 3:**
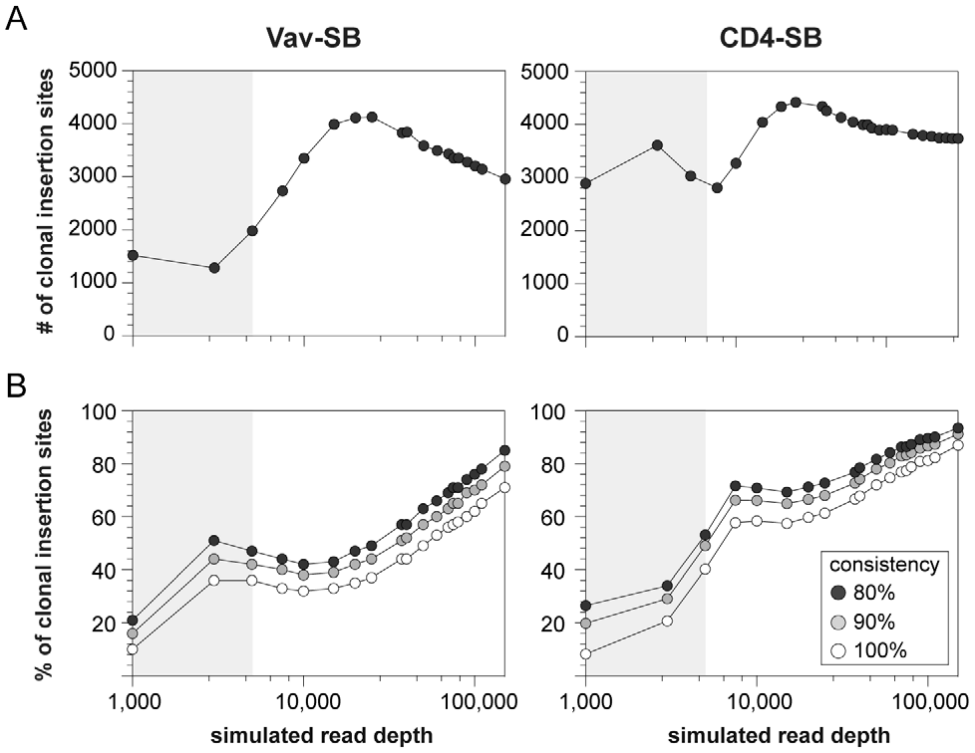
Determining optimal read depth in SB-induced tumors. (A) An experiment was performed to simulate various read depths in 30 Vav-SB (left) and 32 CD4-SB (right) tumors. The average number of clonal transposon insertion sites was determined from 20 independent iterations of each read depth simulation. (B) The consistency of transposon insertion site identification varied with sequence depth. For example, only 10% of transposon insertion sites were identified in 100% simulations at a simulated read depth of 1,000.

We next examined how varying read depth affects the identification of clonal transposon insertion sites to determine the minimum sequence depth required to obtain a consistent result. This was accomplished by random sampling of mapped reads to simulated a variety of read depths ranging from 1,000 to 150,000 reads per sample. The average number of clonal sites found in all tumors for both the Vav-SB and CD4-SB models was determined based on the results from 20 independent simulations across 19 different read depths (Fig. 3). The clonal insertion sites were identified for each sample in all iterations as described above.

Not surprisingly, the average number of clonal insertion sites increases significantly with greater read depths. The results indicate that simulated read depths that approximate 454 sequencing (Fig. 3, gray boxes) identify only ∼65–75% of clonal sites identified at greater read depths. Interestingly, the simulation results for both tumor models suggest that modest increases in read depth initially introduce background insertion sites that are eliminated with the addition of more sequence data (Fig. 3A).

While the average number of clonal sites stabilizes beyond a read depth of 100,000, the composition of the data sets may fluctuate significantly. We next calculated the percentage of sites that were seen in 80, 90, or 100% of the iterations (Fig. 3B). Again, at read depths that approximate 454 sequencing, roughly 40–50% of the clonal sites were found in 90% of the iterations. The consistency improves steadily as sequence depth increases, as expected. Taken together, the simulation experiments indicate that an average of 100,000 mapped reads per sample are required to reproducibly discover clonal insertion events.

### Determining the impact of PCR-bias on LM-PCR results

It has been well established that PCR can introduce significant bias in the amplification of genomic DNA fragments. A number of parameters have been shown to influence the extent to which any fragment will be amplified including fragment length, GC content and secondary structure [15], [16]. Because of this concern, our Illumina-based LM-PCR approach identifies transposon insertion events using four distinct approaches using two different restriction enzymes in an effort to combat the effects of PCR bias. As the goal of our LM-PCR method is to amplify many fragments from complex samples, we examined how extensively PCR bias affects the identification of transposon insertion sites in SB-induced tumors using the Illumina-based LM-PCR method.

Over 6,000 clonal transposon insertion sites were identified in 62 samples, as previously described (Table S1). These sites were identified, in part, by assuming that the read number for each individual junction fragment is correlated to the abundance of the specific insertion event that produced it. However, the read number could also be influenced by PCR bias, the most likely source being fragment length. Therefore, we calculated the size of the *Alu*I and *Nla*III restriction fragments that each clonal insertion site produced based on the mouse reference genome sequence. Next, we determined the percentage of clonal junction fragments that were identified at each length ranging from 20 to 500 bases. These values were chosen because they represent the technical limits to our analysis pipeline.

As shown in Figure S6, there is some preference for amplification of smaller junction fragments, although the effects of PCR bias do not appear to be dramatic. Nevertheless, there is preference for amplification of PCR fragments that are less than 250 bases in length (Figure S6). We then determined the percentage of genomic TA sites (*i.e.* SB target sites) in which all four approaches fail to produce a junction fragment less than 250 bases in length. This analysis indicates that ∼3% of TA sites would not compete well for amplification using our Illumina-based LM-PCR approach — a relatively modest number of sites. Finally, we sought to determine the extent to which the identification of clonal transposon insertion events is affected by PCR bias. This revealed that ∼70% of clonal insertion events were identified as clonal in at least 3 of the 4 libraries generated for each sample (Figure S6). This result suggests that while PCR bias does influence the read number for each insertion site, PCR bias does not greatly impact the identification of clonal insertion sites in tumor samples. However, additional experiments are required to determine if this method provides quantitative information beyond the identification of clonal insertion sites.

### Identification of driver mutations

We have shown that the Illumina-based LM-PCR method significantly outperforms previous methods in its ability to reproducibly identify clonal transposon insertion events within SB-induced tumors. Next, we sought to determine how the Illumina-based LM-PCR method affects the identification of CISs, the functional equivalent of driver mutations in SB-induced tumor models. Previous studies have used one of two methods to identify CISs, Gaussian Kernel Convolution (GKC) or a Monte Carlo (MC) simulation [6], [7], [8], [17]. Both methods assume that background mutations will happen at random throughout the genome and that causative mutations will cluster in regions near or within cancer genes. These regions are statistically defined as those that have a higher mutation rate than expected by chance. A strength of the GKC and MC approaches is that they are relatively unbiased in the identification of CISs. Neither method considers annotated functional elements within the genome, and thus they are well suited to identify novel or poorly characterized genes. However, GKC and MC can also identify CISs that are too small or large to be biologically meaningful, and thus the gene target of some CIS regions is difficult to identify.

Given the limitations of the GKC and MC methods, we developed a novel computational method to identify CIS regions in SB-induced tumors. In contrast to the previous methods, this novel method specifically examines transcribed regions of the genome to identify genes that are mutated at a rate higher than expected by chance. We refer to this method as gene-centric common insertion site (gCIS) analysis. The gCIS method calculates the observed and expected number of insertion events within each RefSeq gene. The expected number of insertion events is based on the number of tumors analyzed, the number of insertion events within each tumor and the number of SB target sites within each RefSeq transcription unit, including 10 kilobases of promoter sequence. These values are used to perform a Chi-square test, yielding a p-value for each RefSeq gene. A Bonferroni correction is applied to correct for multiple hypothesis testing. Any remaining RefSeq genes that were not mutated in at least three independent tumors are also eliminated.

**Figure 4:**
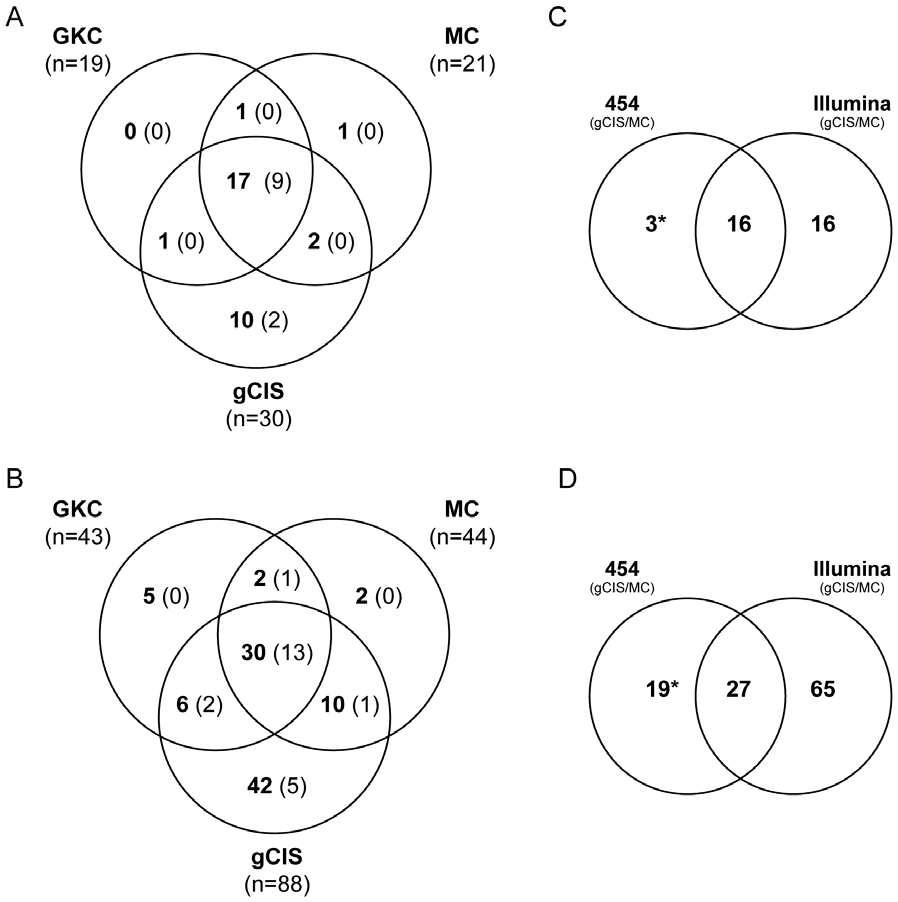
Comparison of three independent methods to identify CISs within SB-induced tumors. We compared the performance of Monte Carlo simulation (MC), Gaussian Kernel Convolution (GKC) and gene-centric common insertion site analysis (gCIS) in identifying candidate cancer genes in both Vav-SB (A) and CD4-SB (B) tumors. In addition, the number in parentheses indicates the number of total genes in each region that have evidence as human cancer genes in either the COSMIC or CGC databases. In addition, we compared the results of MC and gCIS analysis using data generated by 454 or Illumina sequencing of the same tumor samples in both Vav-SB (C) and CD4-SB (D) lymphoma models.

We next determined how each of the three methods performed in analyzing sequence data generated using the Illumina-based LM-PCR method (Fig. 4). This analysis identified a total 32 and 97 CISs in Vav-SB and CD4-SB tumors, respectively. GKC and MC identified similar gene sets in both models. This outcome was expected given the similarity in these computational methods. By contrast, the gCIS method not only identified ∼90% CISs found by GKC and MC, but 1.5 to 2-fold more CISs unique to this approach. The unique CIS genes found by the gCIS approach show a similar degree of overlap with known human cancer genes found in the COSMIC [18] and Cancer Gene Census [19] databases (Fig. 4). This suggests that the gCIS method performs well and is capable of identifying novel CISs that are missed by prior methods (Figure S7). While the GKC and MC methods do not identify many unique CIS regions, the CIS regions called by these methods often contain more insertion events than the corresponding region identified by gCIS analysis. For example, of the 59 genes identified by both MC and gCIS (Fig. 4), MC identified more insertion events within the CIS region than did gCIS in over 30% of cases. While this difference did not affect the identification of these genes, the relative mutation frequencies as determined by each method frequently varied. Thus, there is benefit from analyzing data sets with multiple methods (*e.g.* MC and gCIS).

Finally, we compared the results of CIS analysis that were obtained using either 454 or Illumina sequencing of the same tumor cohort (Fig. 4C,D). The results show that Illumina sequencing identifies ∼2-fold more driver mutations in both lymphoma models. Not surprisingly, 454 sequencing identified a population of CIS genes that were not identified by Illumina sequencing. However, the majority of these genes (21/22) were identified by Illumina sequencing but not identified as a driver mutation using MC or gCIS methods (Table S3).

### Accuracy of CIS identification

**Figure 5:**
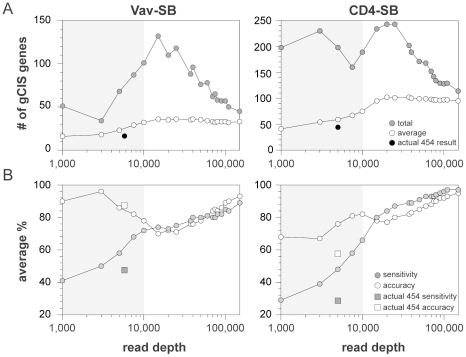
Determining the affect of sequence depth on the accuracy of CIS identification. Read depth simulations were performed as previously described. However, gCIS analysis was performed on each of 20 iterations at all simulated read depths. In addition, the values generated from analysis of the actual 454 data obtained for the same tumor samples are shown. (A) The total number of gCIS genes identified in at least one of the 20 iterations is indicated along with the average number of gCIS genes at each simulated sequence depth. (B) The gCIS results obtained by analyzing all sequence data in both tumor models were used as reference data sets. The accuracy (% of genes found in reference set) and sensitivity (% of genes in reference set that were detected) were determined. For example, only 41% of the reference gCIS genes in the Vav-SB model were found at a depth of 1,000 reads per sample (*i.e.* 41% sensitivity). However, 90% of gCIS genes identified at this read depth were found in the reference data set (*i.e.* 90% accuracy).

Here we have shown that the reproducible identification of transposon insertion sites requires ∼20-fold more sequence data than is currently used to characterize SB-induced tumors (Fig. 3). We predicted that the insufficient sequence coverage would reduce the accuracy of CIS identification. To test this hypothesis, we again performed simulations in which random sequences were selected to mimic various read depths. Twenty iterations were performed for each sequence depth, and gCIS analysis was performed on the data set generated for each iteration. Next, we calculated the total and average number of genes that were called at each sequence depth (Fig. 5A). Consistent with the results of the previous simulation experiment, the gCIS results varied widely at intermediate sequence depths. However, as sequence depth increased, the gCIS results became more consistent.

This result suggested that the accuracy of the gCIS method at low to intermediate sequence depths is likely lower than anticipated. We next calculated the accuracy of gCIS identification using the simulation results. The prior simulation results indicated that sequence depths >100,000 reads per sample provide consistent gCIS results (Fig. 5A). Therefore, we defined the gCIS gene set identified using all sequence data as the reference data set. The average sensitivity and accuracy of gCIS analysis was then determined by comparing the results obtained for each iteration and sequence depth to the reference data set (Fig. 5B). As predicted, the sensitivity of gCIS detection is relatively poor at low sequence depths. This was confirmed by comparing the results of the simulation to the actual data obtained using 454 sequencing (Fig. 5). For the most part, the 454 results were similar to the predicted outcome based on the sequence depth simulations, although the actual 454 data consistently performed worse than predicted.

**Figure 6:**
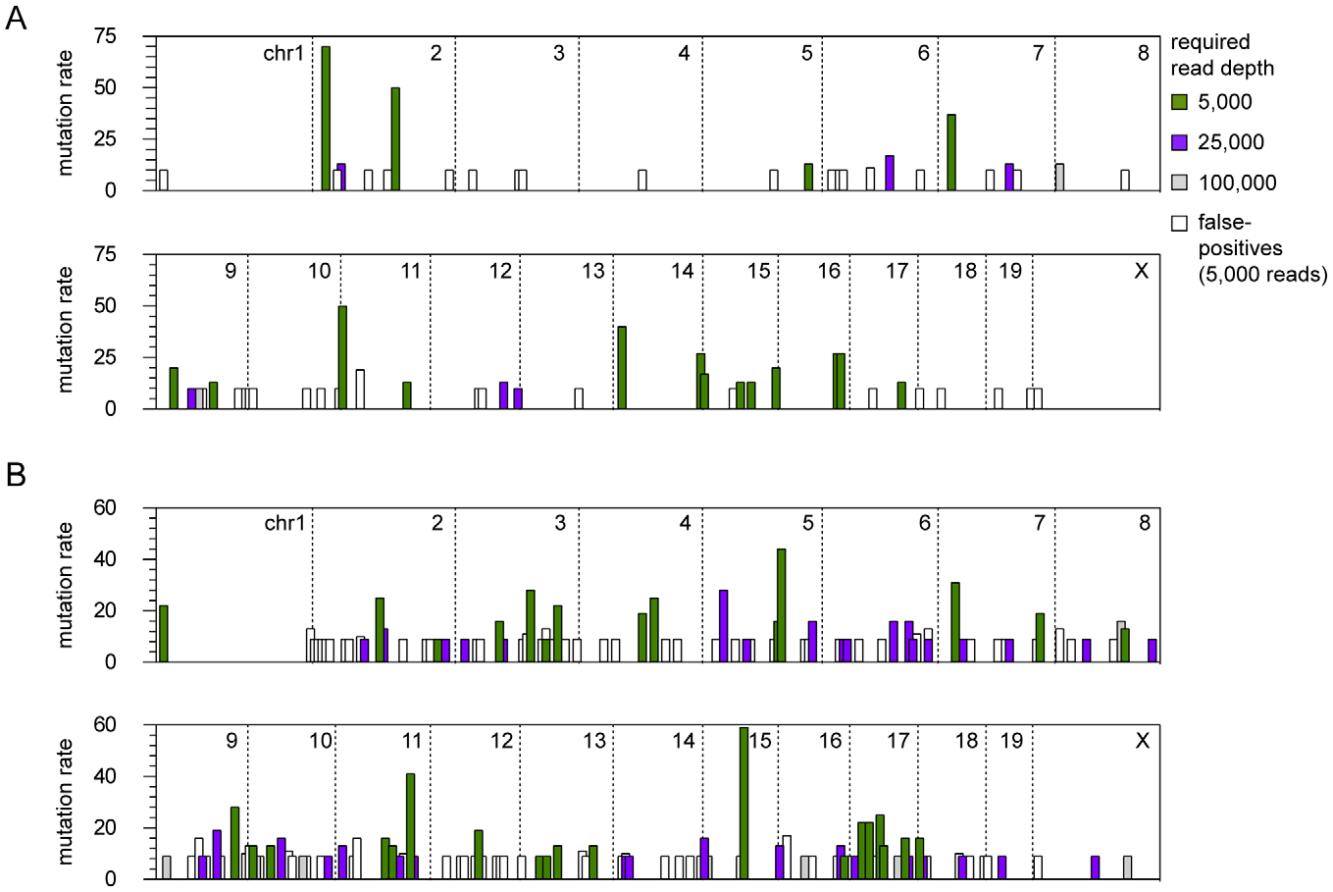
The affect of varying read depth on the genomic distribution of CIS genes. The genomic position of each CIS gene is indicated as a vertical bar. The color of the bar indicates the minimum number of reads per sample required to consistently identify the CIS gene at the indicated position. The height of the bar indicates the mutation frequency within the each tumor model as determined by analysis of all sequence data. A large number of false-positive CISs were identified in one or more iterations of the simulation to approximate a read depth of 5,000 (white bars).

In general, the gCIS analysis performed much better across a range of sequence depths in Vav-SB tumors. We have previously shown that the CD4-SB tumors show greater genetic complexity than Vav-SB tumors, harboring more mutations per sample that affect a larger number of genes [20]. As a consequence, the accuracy and sensitivity of gCIS identification in CD4-SB tumors are more significantly affected by inadequate sequence coverage. The distinction between the Vav-SB and CD4-SB tumor models is clearer when the genomic distributions of driver mutations are compared (Fig. 6). Not surprisingly, the majority of CIS genes in the Vav-SB model can be identified at low to moderate sequence depths. By contrast, the identification of many CIS genes in the CD4-SB model requires additional sequence data. This result indicates that tumor complexity in SB-induced models of cancer will determine the needed sequence depth to consistently identify CIS genes. It is also important to note that increased sequence depths are required to identify and eliminate false-positive CIS genes regardless of tumor complexity. For example, the majority of CIS regions identified at a read depth of 5,000 reads per sample are eliminated with the addition of more sequence data (Fig. 6, white bars). In this regard, the Illumina-based LM-PCR method would likely reduce the false-discovery rate of CIS identification, assuming sufficient sequence coverage is obtained for each sample.

## Discussion

As with the identification of somatic mutation in human tumors, the analysis of transposon-induced mutations in SB models of cancer relies on the sensitivity to detect mutations within heterogeneous tumor cell populations. The Illumina-based LM-PCR method we describe here shows a marked improvement in sensitivity — increasing the average sample read depth by ∼50-fold (Table S1). This increase in sequence coverage led to a 15-fold increase in the average number of mapped transposon insertion sites per sample.

The sheer number of insertion sites identified per sample (>15,000) provides clear evidence that the Illumina-based LM-PCR is capable of identifying rare transposon insertion events likely present in a small percentage of cells. These rare insertion events significantly outnumber the clonally expanded insertion events that are also present in each tumor sample (Fig. 1). Their inclusion in the data set confounds efforts to identify CIS genes in these tumors cohorts. Therefore, we devised three independent methods to identify and remove such background sites prior to CIS analysis. Our analysis shows that not only that these background insertion sites must be removed, but that multiple methods are required for this process (Fig. 1B). For example, the CIS genes identified using the dynamic cutoff method showed the greatest overlap with genes implicated in human cancer (Table S2).

The need for such a dynamic cutoff method is likely caused by heterogeneity within SB-induced tumors. Transposon copy number has been shown to affect tumor latency and the complexity of transposon insertion sites [6], [9]. The Vav-SB and CD4-SB tumors were generated using high copy transposon donor strains [11]. Therefore, these tumors typically harbor a greater number of clonal transposon insertion events when compared to similar tumors generated using low copy transposon donor strains. With this knowledge, our goal was to develop an analysis pipeline that is more dynamic, allowing it to process data derived from a variety of transposon strains without further optimization. Thus the dynamic cutoff method was employed.

Another benefit of increased sequence depth is that it provided the opportunity to simulate the effects of varying sequence coverage on all aspects of insertion site analysis. Prior publications have relied on 454 sequencing to identify transposon-induced mutations in SB models of cancer. We have shown that ∼20-fold increase in sequence depth over what is achieved with 454 is required to eliminate sampling error and consistently identify clonal transposon insertion events. Increased sequence coverage also provided more consistent results in the identification of CIS genes.

The Illumina-based LM-PCR approach we describe here is clearly superior to the prior 454-based method that we and others have used to characterize SB-induced tumors. However, we have also shown that the results of this approach are influenced by PCR bias. However, the effects of PCR bias are mostly offset by employing four different approaches to identify insertion sites. It should be noted that it is not possible to infer any additional quantitative information from the Illumina-based sequencing method, aside from the identification of clonal transposon insertion sites, given the persistent PCR bias observed. Likely this bias is caused by the continued use of restriction enzymes to fragment the genomic DNA. Alternative approaches such as non-restrictive LAM-PCR [21] or the use of physical shearing to fragment the genome could eliminate the PCR bias we observed. Regardless, additional work is required to establish a truly quantitative approach to identify transposon insertion sites in SB-induced tumor samples. This type of quantitative method will likely be needed to more specifically identify transposon insertion sites that have been clonally expanded in tumor cell populations.

In addition to the Illumina-based LM-PCR method, we also describe a novel computational method to identify CIS genes in SB-induced tumors. We show that the gene-centric CIS (gCIS) method is capable of identifying a novel set of genes that are missed by established methods (*e.g.* MC, GKC). A subset of the gCIS-specific genes are also mutated in human cancer, suggesting that this approach will be useful in extracting additional genetic information from SB-induced tumors. Another advantage of the gCIS method is that it provides an individual assessment of all RefSeq genes in the mouse genome, while the MC and GKC methods are less specific in the identification of driver mutations. As a consequence, the gCIS method provides an output that will facilitate comparative oncogenomic approaches using genetic data derived from human tumors. Thus the gCIS method complements the exiting computation approaches currently in use.

Rapid improvements in DNA sequencing technology have now made tumor genome sequencing a reality. However, early efforts in this area suggest that while sequencing the human cancer genome is achievable, our ability to understand the biological significance of each somatically acquired mutation within an individual's tumor is somewhat limited. Largely this is due to our inability to accurately identify driver mutations within the larger field of passenger mutations that coexist within each tumor. Thus, additional experimental evidence will be needed to assess the potential of each candidate driver mutation that is identified by the tumor genome sequencing.

Genetically engineered mouse models have been used to study a variety of cancer-relevant phenotypes. In addition, mouse models of cancer have been exploited to identify common genetic mechanisms that drive both mouse and human cellular transformation. More recently, *in vivo* insertional mutagenesis has been used to generate mouse models of cancer in which the identification of candidate cancer genes is dramatically accelerated [6], [7], [12], [22], [23]. Recent work has demonstrated the advantage of using insertional mutagenesis to study a variety of cancer-relevant phenotypes such as metastasis and acquired drug resistance in mouse models of cancer [7], [24]. The method we describe here will improve the quality of data and provide greater confidence in the identification of candidate cancer genes in these SB models of cancer. Ultimately, our work will improve the ability to use SB models to perform comparative oncogenomics to assist in deciphering the human cancer genome.

## Methods

### Tumor cohort

The Vav-SB and CD4-SB mouse models are described previously [20]. All tumors used in this study were collected from mice using procedures approved and monitored by the Institutional Animal Care and Use Committee at the University of Iowa.

### Read Analysis

Reads were analyzed using the Integration Analysis System (IAS). This pipeline was run independently on each lane. The inputs to the pipeline were a FASTA file containing all the sequencing reads and a barcode file that per barcode contains: the barcode sequence, the name for the tumor, IRL or IRR (to indicate which from which inverted repeat the sequence is derived), and the expected flanking sequences. Using the barcode, each sequence was placed into a tumor specific file. Crossmatch (P. Green, unpublished) identifies the presence and location of the flanking sequences. This information is used to trim the flanking sequences from the genomic segment. All sequences were then verified to begin with a “TA” dinucleotide. The genomic sequences were then aligned to the mouse genome reference assembly (NCBI37/mm9) using Bowtie [13]. The parameters used were “–best -f -k2 –p7 –v3” which gives the top two hits and only allows 3 mismatches in the sequence. The Bowtie output was then filtered using the following criteria: the best match had to be at least 90% identical, including a perfect match to the “TA” at the start of the alignment, be at least 5% better than the second best match, and have 2 or less mismatches. Using the refFLat table from the UCSC genome databases [25], we retrieved the gene-centric data with which to annotate the integrations. The annotation file included the following information: [tumor ID], [gene name], [gene region hit (*i.e.* intron, exon)], [predicted affect of transposon insertion on gene expression], [distance from gene], [chromosome], [address], [percent of reads derived from the insertion site], and [transposon orientation relative the gene].

After each annotation file is generated, all transposon insertion events that map to the same chromosome as the transposon donor transgene were removed. This was done to eliminate any bias caused by local hopping of SB transposons. Next, the clonality cutoff was calculated for each annotation file. The clonal insertion sites identified in each of the four LM-PCR libraries generated for each sample (Figure S1) were then combined to generate a non-redundant list of insertion sites identified in each sample. In cases where an insertion site was identified in multiple libraries, the largest value representing the percent of reads derived from the insertion site is used.

### Identifying clonal insertion sites

Three methods were used to identify clonal insertion events within each tumor sample. All three methods are preformed independently on the reads obtained for each barcode, and the maximum cutoff value is applied. The negative binomial models the background distribution. The background is estimated by fitting a negative binomial distribution to the distribution of sites with 1–3 reads. These sites were selected to model the background because we are confident that these sites are not clonally expanded. The number of reads was zero shifted to better approximate the negative binomial. The dnbinom module in R was then used to generate the negative binomial distribution. The best fit to the negative binomial distribution was determined by minimizing the least squared error across all iterations of the size and probability parameters in steps of 0.01 and 0.001, respectively. The NB threshold was calculated as the read depth at which 95% of the integration sites are under the curve. Thus only 5% of the background distribution is expected to contribute to the set of clonal sites. A second cutoff value was calculated by taking 1% of the read number observed for the most abundant site. The third cutoff value was defined as the value representing 0.1% of the total reads for the barcode.

### Identifying Causative Genes

The Monte Carlo simulation and Gaussian Kernel Convolution were performed essentially as previously published [6], [17]. Gene-centric CISs (gCIS) were defined based upon the number of TA dinucleotides within the transcribed region for each gene in the RefSeq collection. The gene-associated region was defined as the union of all RefSeq transcripts including a 10 kb promoter region. A chi-squared test statistic was calculated based upon the number of TAs within each gene-associated region and the number of integrations within the gene. This test statistic was used to determine the p-value, with a single degree of freedom. A tab-delimited file was generated detailing the results for each RefSeq gene including the gCIS p-value, number of tumors in which integrations were found, and a list of the tumor IDs in which integrations were observed. Using the Bonferroni method, a statistical threshold of p = 2.63×10−6 was used to correct for multiple hypothesis testing. A final condition applied to all methods was a requirement for each CIS to harbor a minimum of three independent tumors with insertions in the gene.

### Simulation of read depth

To simulate read depth, we randomly sampled with replacement mapped reads from a pooled set. This set contained the map location of the read and corresponding tumor ID from which it was identified. Twenty iterations of each read depth were generated, and the average number of clonal insertion sites was calculated for each tumor model. The consistency of insertion site identification was calculated by calculating the percentage of insertion sites that were identified in 80, 90 or 100% of the iterations for each read depth. In addition, gCIS analysis was performed on each of the twenty iterations at each read depth. These results were then compared the reference gCIS data set (*i.e.* true positives) obtained by analyzing all sequence data. Thus for each simulated read depth, gCIS accuracy is the percentage of gCIS genes found at each read depth that are also present in the reference data set. The gCIS sensitivity is the percentage of gCIS genes in the reference data set that is found at each simulated read depth.

### Comparison to human cancer genes

A comparative oncogenomics approach was used to assess the extent to which CIS analysis identifies genes implicated in human cancer. This was done by comparing individual CIS genes are mutated in the COSMIC and Cancer Gene Census databases [18], [19]. While the Cancer Gene Census contains only validated cancer genes, the COSMIC database contains data on more than 19,000 genes. Somatic mutations have been identified in only a subset of these genes. We generated a list of COSMIC genes that have a mutation frequency of at least 5% with a minimum of 5 independent somatic mutations. Ultimately, we identified 807 genes that have supporting evidence of somatic mutation in human tumors and clear mouse orthologs.

## Supporting Information

Figure S1 **Overview of ligation-mediated PCR strategy to amplify transposon-genomic junctions from SB-induced tumors.** Each tumor DNA is digested with either *Alu*I or *Nla*III restriction enzyme. These enzymes create junction fragments on both ends of each integrated transposon. Double-stranded adaptors are then ligated to the ends of all genomic DNA fragments. The adaptor is modified to such that the adaptor primer used in the primary PCR reaction cannot hybridize until the transposon-specific primer first generates the complementary strand. Nested PCR is then performed using a primers modified to include the sequence tags required for direct sequencing on the Illumina platform.
(PDF)

Figure S2 **(A) Overview of Sleeping Beauty (SB) models of cancer.** (B) We used a Cre-inducible SB transposon mutagenesis system to generate two distinct models of T-cell lymphoma in which mutagenesis was initiated in either hematopoietic stem cells (Vav-SB) or in nearly differentiated thymocytes (CD4-SB). (C) Tumors within Vav-SB and CD4-SB mice develop with different latencies and are driven by distinct mutations. The major findings of this work are described elsewhere [20].
(PDF)

Figure S3 **An aliquot of the secondary LM-PCR product (25 µl of a 100 µl reaction) is analyzed by agarose gel electrophoresis to verify the quality of the sample. The above example is a typical result for the LM-PCR process.** Products typically appear as a low molecular weight smear, although some samples have more abundant junction products that appear as bands. Failed reactions may have only a primer dimer band of ∼100 bp. The LM-PCR process is repeated on these samples until a similar result is obtained. [DNA marker is the 1 kb+ ladder (Invitrogen). Indicated bands are base pair values.]
(PDF)

Figure S4 **Overview of sequence analysis pipeline.** The raw Illumina sequence file (FASTQ formatted) and the barcode file containing the metadata for each tumor sample are the inputs for the analysis pipeline. The order and a brief description for each stepwise process is shown.
(PDF)

Figure S5 **The read distribution is shown for two different samples that were analyzed by both Illumina (A) and 454 (B) LM-PCR.** The increased sequence depth in the Illumina method apparently improves the signal to noise ratio and identifies more clonal insertion sites than 454 sequencing.
(PDF)

Figure S6 **Effects of PCR bias on the results of Illumina-based LM-PCR.** The average restriction fragment sizes generated by clonal transposon insertions in CD4-SB (A) or Vav-SB tumors (B) is shown. This clearly shows preferential amplification of smaller junction fragments as they are more abundant than expected given the size distribution in the genome (shown in red). However, this trend appears to be similar in junction fragments that were identified as clonal (white circles) or subclonal (gray circles) transposon insertion sites. (C) Based on the range of product sizes that are preferentially amplified (A–B, gray box), we determined the percentage of genomic TA sites that would be subject to negative PCR bias in all four libraries. While PCR bias is evident, it does not appear to dramatically affect the identification of clonal transposon insertion sites. (D) The majority of clonal sites were identified as clonal in at least three of the four junction libraries generated for each tumor.
(PDF)

Figure S7 **The gene-centric common insertion site (gCIS) approach was designed to identify genes that suffer transposon-induced mutations at a rate higher than expected given the number of tumors, number of insertion sites per tumor and the number SB target sites in each gene.** This method is capable of identifying driver mutations that are not identified by existing methods (MC, GKC) that rely on transposon clustering to identify common insertion sites. As a consequence, diffuse clusters of insertion events in some genes are missed by these methods. By contrast, the gCIS method can identify these genes as significant driver mutations. Shown here are three such examples. In each case, MC failed to identify the gene as a driver mutations while gCIS identified it as a candidate cancer gene. The general structure is shown for each gene (vertical lines = exons, angled lines = spliced introns) including arrows indicating the position of transposon insertions (green arrow = forward transposon orientation, red arrow = reverse transposon orientation).
(PDF)

Table S1 **Summary analysis using 454 and Illumina-based LM-PCR.**
(PDF)

Table S2 **Comparison of cutoff methods in the identification of CIS and gCIS genes.**
(PDF)

Table S3 **Comparison of CIS genes identified by 454 and Illumina sequencing in SB-induced lymphomas.**
(PDF)

Methods S1 **Detailed Illumina-based LM-PCR protocol.**
(PDF)
