# Supplementary material for: Novel Molecular and Computational Methods Improve the Accuracy of Insertion Site Analysis in Sleeping Beauty-Induced Tumors
Source: PLoS One. 2011 Sep 13;6(9):e24668. doi: 10.1371/journal.pone.0024668 (PMC3172244; doi:10.1371/journal.pone.0024668)
Supplement: Figure S1 — Overview of ligation-mediated PCR strategy to amplify transposon-genomic junctions from SB-induced tumors. Each tumor DNA is digested with either AluI or NlaIII restriction enzyme. These enzymes create junction fragments on both ends of each integrated transposon. Double-stranded adaptors are then ligated to the ends of all genomic DNA fragments. The adaptor is modified to such that the adaptor primer used in the primary PCR reaction cannot hybridize until the transposon-specific primer first generates the complementary strand. Nested PCR is then performed using a primers modified to include the sequence tags required for direct sequencing on the Illumina platform. (PDF) [file pone.0024668.s001.pdf]

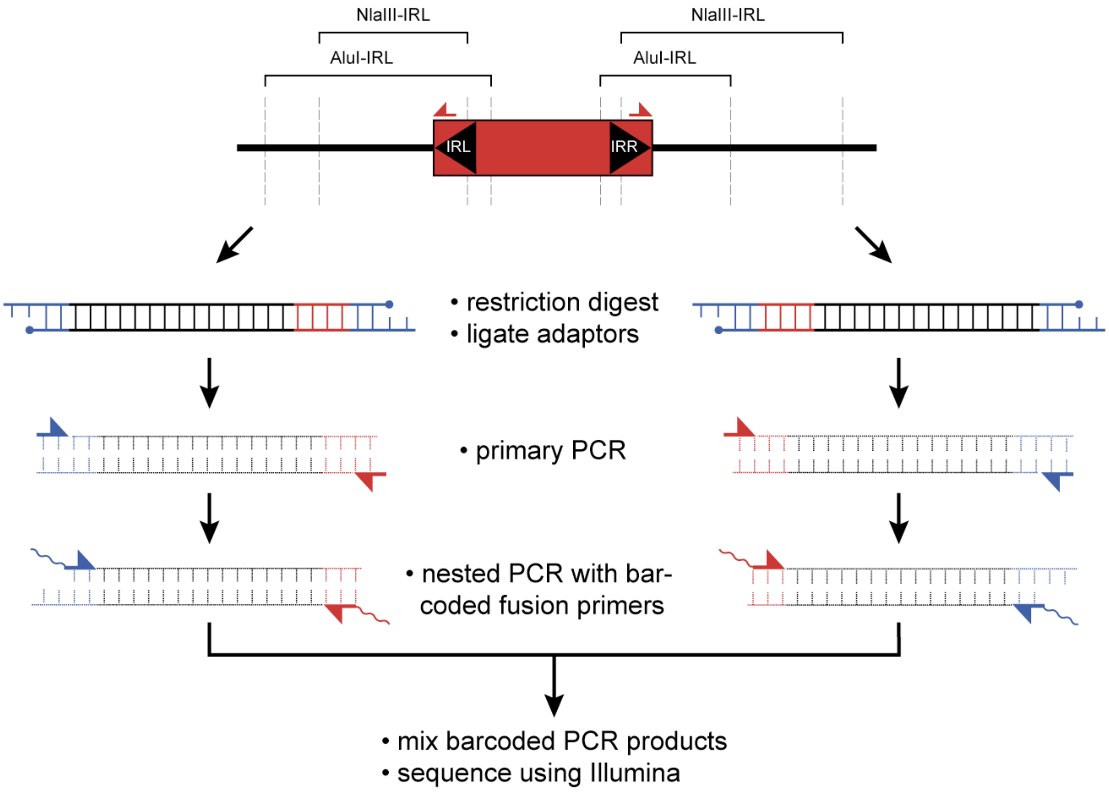


**Figure S1.** Overview of ligation-mediated PCR strategy to amplify transposon-genomic junctions from SB-induced tumors. Each tumor DNA is digested with either *Alu*I or *Nla*III restriction enzyme. These enzymes create junction fragments on both ends of each integrated transposon. Double-stranded adaptors are then ligated to the ends of all genomic DNA fragments. The adaptor is modified to such that the adaptor primer used in the primary PCR reaction cannot hybridize until the transposon-specific primer first generates the complementary strand. Nested PCR is then performed using a primers modified to include the sequence tags required for direct sequencing on the Illumina platform.
