# Supplementary material for: Novel Molecular and Computational Methods Improve the Accuracy of Insertion Site Analysis in Sleeping Beauty-Induced Tumors
Source: PLoS One. 2011 Sep 13;6(9):e24668. doi: 10.1371/journal.pone.0024668 (PMC3172244; doi:10.1371/journal.pone.0024668)
Supplement: Figure S2 — (A) Overview of Sleeping Beauty (SB) models of cancer. (B) We used a Cre-inducible SB transposon mutagenesis system to generate two distinct models of T-cell lymphoma in which mutagenesis was initiated in either hematopoietic stem cells (Vav-SB) or in nearly differentiated thymocytes (CD4-SB). (C) Tumors within Vav-SB and CD4-SB mice develop with different latencies and are driven by distinct mutations. The major findings of this work are described elsewhere [20]. (PDF) [file pone.0024668.s002.pdf]

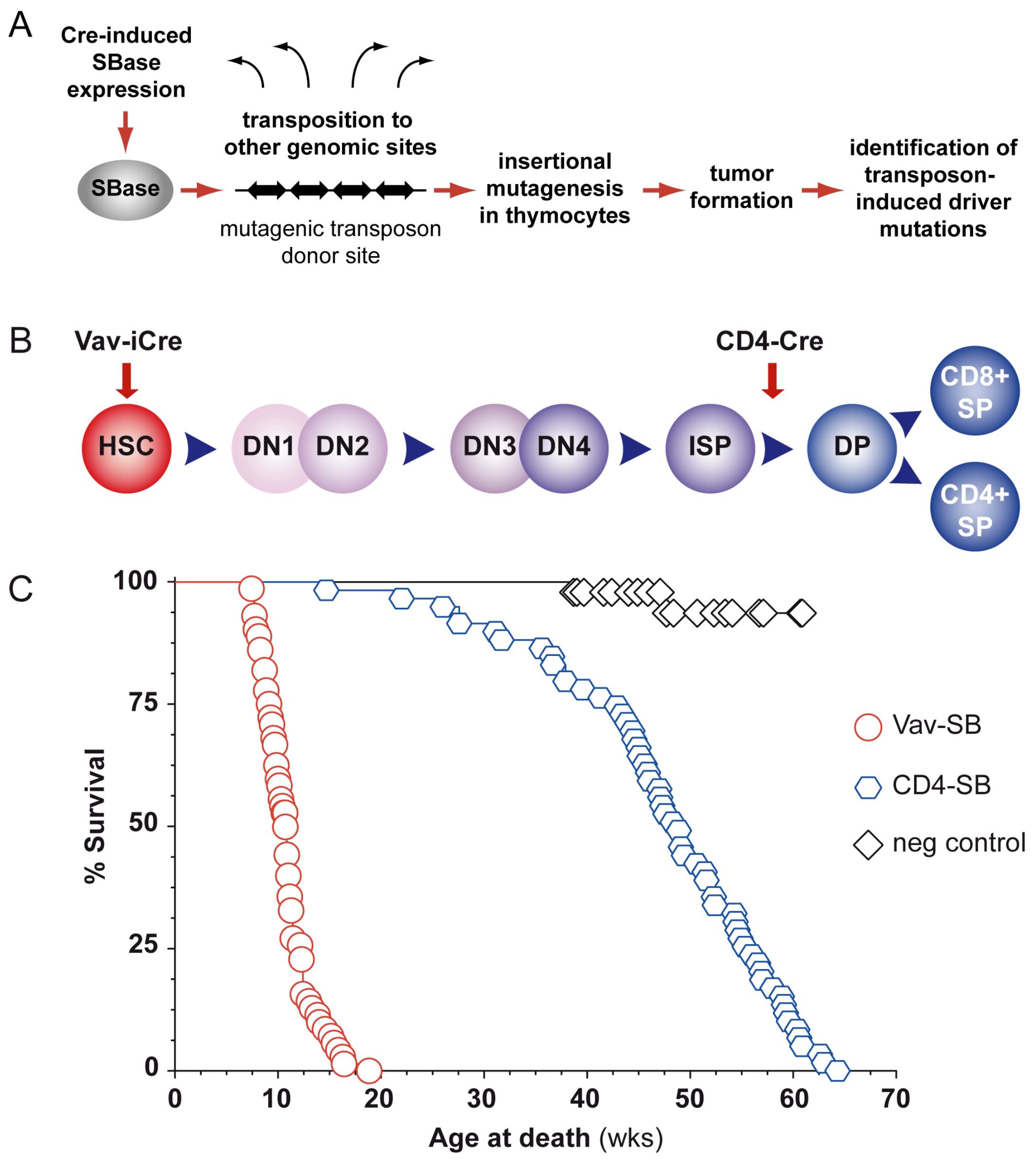

Figure S2. (A) Overview of Sleeping Beauty (SB) models of cancer. (B) We used a Cre-inducible SB transposon mutagenesis system to generate two distinct models of T-cell lymphoma in which mutagenesis was initiated in either hematopoietic stem cells (Vav-SB) or in nearly differentiated thymocytes (CD4-SB). (C) Tumors within Vav-SB and CD4-SB mice develop with different latencies and are driven by distinct mutations. The major findings of this work are described elsewhere (Berquam-Vrieze et al., submitted).
