# Supplementary material for: Novel Molecular and Computational Methods Improve the Accuracy of Insertion Site Analysis in Sleeping Beauty-Induced Tumors
Source: PLoS One. 2011 Sep 13;6(9):e24668. doi: 10.1371/journal.pone.0024668 (PMC3172244; doi:10.1371/journal.pone.0024668)
Supplement: Figure S3 — An aliquot of the secondary LM-PCR product (25 µl of a 100 µl reaction) is analyzed by agarose gel electrophoresis to verify the quality of the sample. The above example is a typical result for the LM-PCR process. Products typically appear as a low molecular weight smear, although some samples have more abundant junction products that appear as bands. Failed reactions may have only a primer dimer band of ∼100 bp. The LM-PCR process is repeated on these samples until a similar result is obtained. [DNA marker is the 1 kb+ ladder (Invitrogen). Indicated bands are base pair values.] (PDF) [file pone.0024668.s003.pdf]

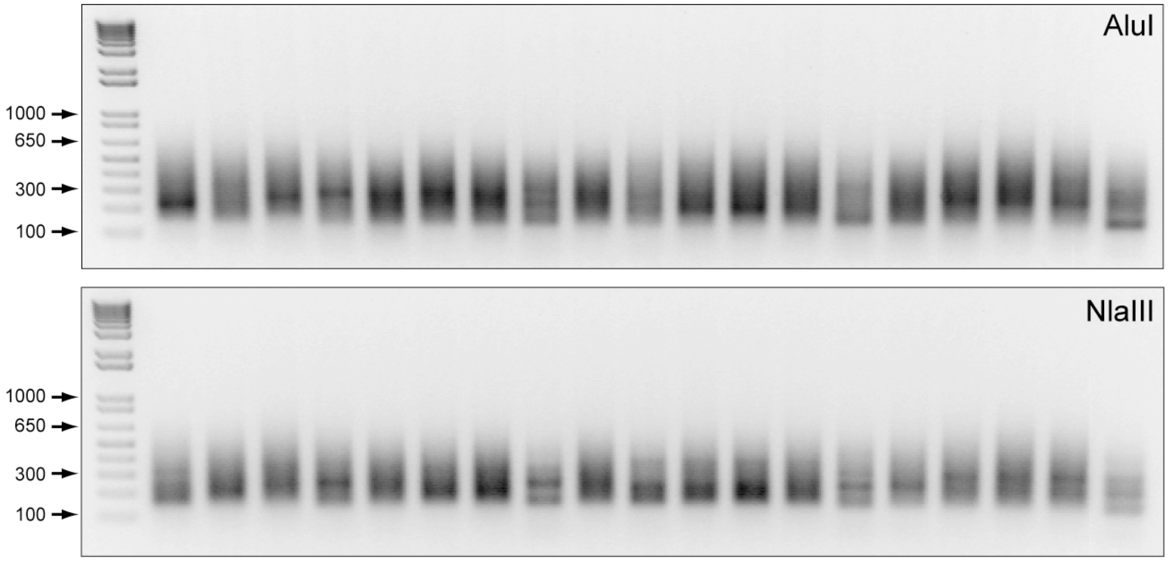

Figure S3. An aliquot of the secondary LM-PCR product (25 μl of a 100 μl reaction) is analyzed by agarose gel electrophoresis to verify the quality of the sample. The above example is a typical result for the LM-PCR process. Products typically appear as a low molecular weight smear, although some samples have more abundant junction products that appear as bands. Failed reactions may have only a primer dimer band of ~100 bp. The LM-PCR process is repeated on these samples until a similar result is obtained. [DNA marker is the 1 kb+ ladder (Invitrogen). Indicated bands are base pair values.]
