# Supplementary material for: Novel Molecular and Computational Methods Improve the Accuracy of Insertion Site Analysis in Sleeping Beauty-Induced Tumors
Source: PLoS One. 2011 Sep 13;6(9):e24668. doi: 10.1371/journal.pone.0024668 (PMC3172244; doi:10.1371/journal.pone.0024668)
Supplement: Figure S4 — Overview of sequence analysis pipeline. The raw Illumina sequence file (FASTQ formatted) and the barcode file containing the metadata for each tumor sample are the inputs for the analysis pipeline. The order and a brief description for each stepwise process is shown. (PDF) [file pone.0024668.s004.pdf]

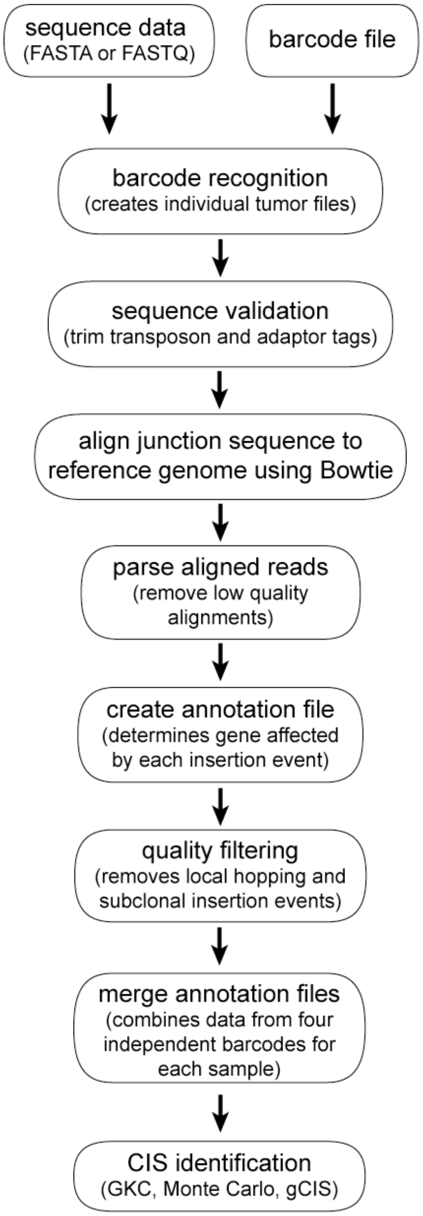

Figure S4. Overview of sequence analysis pipeline. The raw Illumina sequence file (FASTQ formatted) and the barcode file containing the metadata for each tumor sample are the inputs for the analysis pipeline. The order and a brief description for each stepwise process is shown.
