# Supplementary material for: Novel Molecular and Computational Methods Improve the Accuracy of Insertion Site Analysis in Sleeping Beauty-Induced Tumors
Source: PLoS One. 2011 Sep 13;6(9):e24668. doi: 10.1371/journal.pone.0024668 (PMC3172244; doi:10.1371/journal.pone.0024668)
Supplement: Figure S5 — The read distribution is shown for two different samples that were analyzed by both Illumina (A) and 454 (B) LM-PCR. The increased sequence depth in the Illumina method apparently improves the signal to noise ratio and identifies more clonal insertion sites than 454 sequencing. (PDF) [file pone.0024668.s005.pdf]

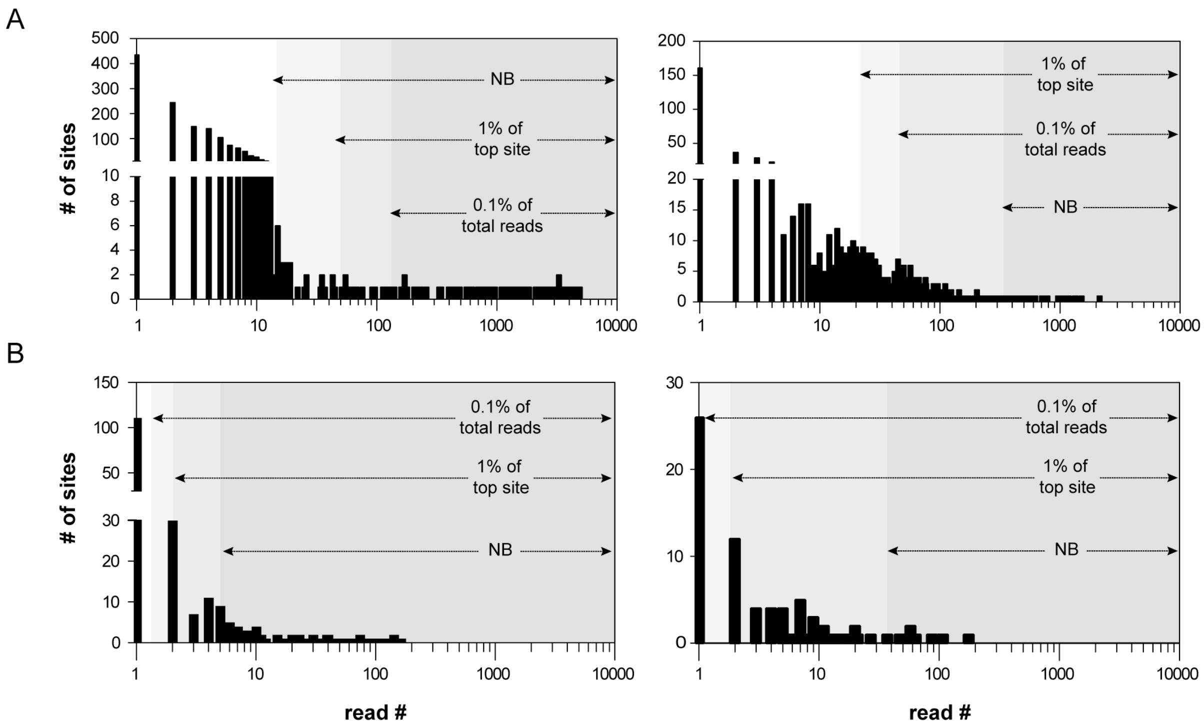

Figure S5. The read distribution is shown for two different samples that were analyzed by both Illumina (A) and 454 (B) LM-PCR. The increased sequence depth in the Illumina method apparently improves the signal to noise ratio and identifies more clonal insertion sites than 454 sequencing.
