# Supplementary material for: Novel Molecular and Computational Methods Improve the Accuracy of Insertion Site Analysis in Sleeping Beauty-Induced Tumors
Source: PLoS One. 2011 Sep 13;6(9):e24668. doi: 10.1371/journal.pone.0024668 (PMC3172244; doi:10.1371/journal.pone.0024668)
Supplement: Figure S6 — Effects of PCR bias on the results of Illumina-based LM-PCR. The average restriction fragment sizes generated by clonal transposon insertions in CD4-SB (A) or Vav-SB tumors (B) is shown. This clearly shows preferential amplification of smaller junction fragments as they are more abundant than expected given the size distribution in the genome (shown in red). However, this trend appears to be similar in junction fragments that were identified as clonal (white circles) or subclonal (gray circles) transposon insertion sites. (C) Based on the range of product sizes that are preferentially amplified (A–B, gray box), we determined the percentage of genomic TA sites that would be subject to negative PCR bias in all four libraries. While PCR bias is evident, it does not appear to dramatically affect the identification of clonal transposon insertion sites. (D) The majority of clonal sites were identified as clonal in at least three of the four junction libraries generated for each tumor. (PDF) [file pone.0024668.s006.pdf]

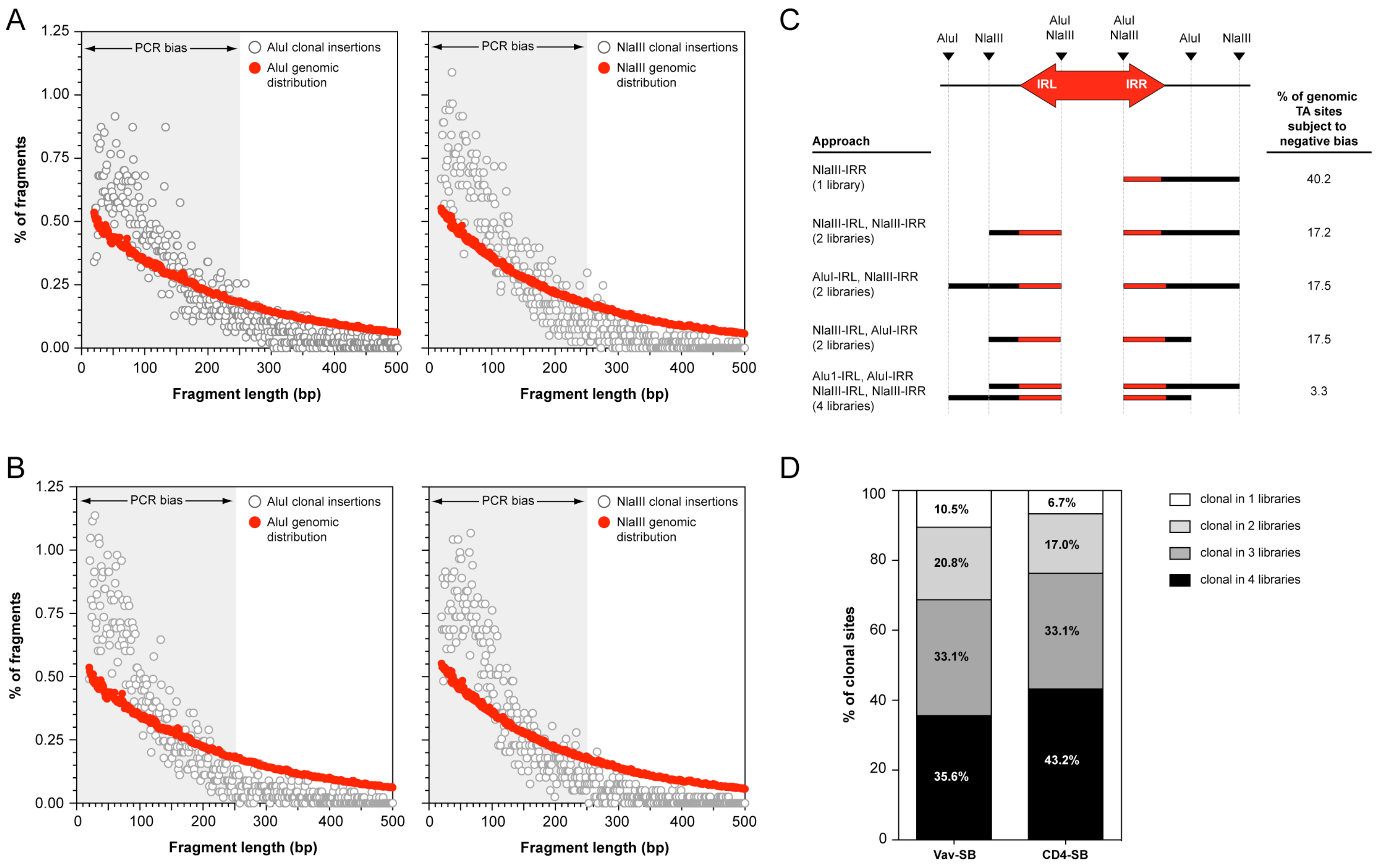

Figure S6. Effects of PCR bias on the results of Illumina-based LM-PCR. The average restriction fragment sizes generated by clonal transposon insertions in CD4-SB (A) or Vav-SB tumors (B) is shown. This clearly shows preferential amplification of smaller junction fragments as they are more abundant than expected given the size distribution in the genome (shown in red). However, this trend appears to be similar in junction fragments that were identified as clonal (white circles) or subclonal (gray circles) transposon insertion sites. (C) Based on the range of product sizes that are preferentially amplified (A-B, gray box), we determined the percentage of genomic TA sites that would be subject to negative PCR bias in all four libraries. While PCR bias is evident, it does not appear to dramatically affect the identification of clonal transposon insertion sites. (D) The majority of clonal sites were identified as clonal in at least three of the four junction libraries generated for each tumor.
