# Supplementary material for: Novel Molecular and Computational Methods Improve the Accuracy of Insertion Site Analysis in Sleeping Beauty-Induced Tumors
Source: PLoS One. 2011 Sep 13;6(9):e24668. doi: 10.1371/journal.pone.0024668 (PMC3172244; doi:10.1371/journal.pone.0024668)
Supplement: Figure S7 — The gene-centric common insertion site (gCIS) approach was designed to identify genes that suffer transposon-induced mutations at a rate higher than expected given the number of tumors, number of insertion sites per tumor and the number SB target sites in each gene. This method is capable of identifying driver mutations that are not identified by existing methods (MC, GKC) that rely on transposon clustering to identify common insertion sites. As a consequence, diffuse clusters of insertion events in some genes are missed by these methods. By contrast, the gCIS method can identify these genes as significant driver mutations. Shown here are three such examples. In each case, MC failed to identify the gene as a driver mutations while gCIS identified it as a candidate cancer gene. The general structure is shown for each gene (vertical lines = exons, angled lines = spliced introns) including arrows indicating the position of transposon insertions (green arrow = forward transposon orientation, red arrow = reverse transposon orientation). (PDF) [file pone.0024668.s007.pdf]

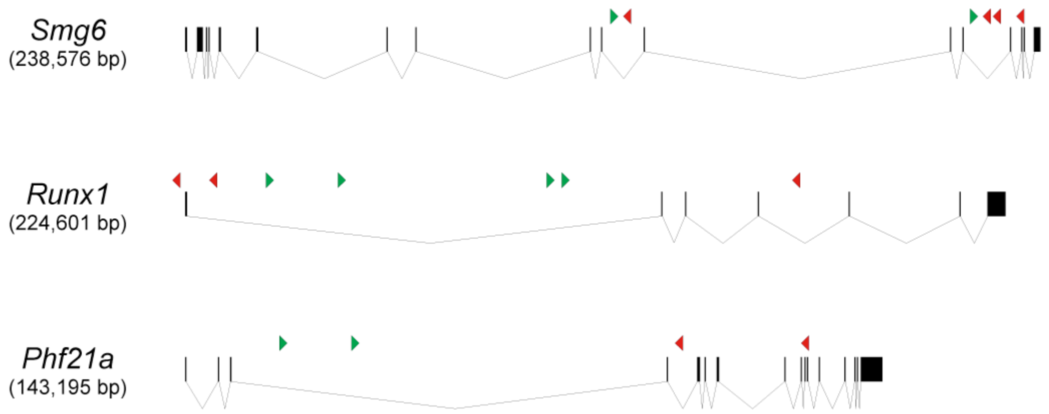

Figure S7. The gene-centric common insertion site (gCIS) approach was designed to identify genes that suffer transposon-induced mutations at a rate higher than expected given the number of tumors, number of insertion sites per tumor and the number SB target sites in each gene. This method is capable of identifying driver mutations that are not identified by existing methods (MC, GKC) that rely on transposon clustering to identify common insertion sites. As a consequence, diffuse clusters of insertion events in some genes are missed by these methods. By contrast, the gCIS method can identify these genes as significant driver mutations. Shown here are three such examples. In each case, MC failed to identify the gene as a driver mutations while gCIS identified it as a candidate cancer gene. The general structure is shown for each gene (vertical lines = exons, angled lines = spliced introns) including arrows indicating the position of transposon insertions (green arrow = forward transposon orientation, red arrow = reverse transposon orientation).
