# Supplementary material for: Novel Molecular and Computational Methods Improve the Accuracy of Insertion Site Analysis in Sleeping Beauty-Induced Tumors
Source: PLoS One. 2011 Sep 13;6(9):e24668. doi: 10.1371/journal.pone.0024668 (PMC3172244; doi:10.1371/journal.pone.0024668)
Supplement: Table S1 — Summary analysis using 454 and Illumina-based LM-PCR. (PDF) [file pone.0024668.s008.pdf]

Supplemental Table S1. Summary analysis using 454 and Illumina-based LM-PCR

**Model**	**# of samples**	**Illumina**				**454**			
		reads		insertion sites		reads		insertion sites	
		**total**	**avg**	**total**	**clonal**	**total**	**avg**	**total**	**clonal**
Vav-SB	30	8,381,780	279,393	517,027	2,552	174,753	5,825	30,088	1,425
CD4-SB	32	8,057,748	251,805	497,437	3,650	160,382	5,012	36,097	2,729
