# Supplementary material for: Novel Molecular and Computational Methods Improve the Accuracy of Insertion Site Analysis in Sleeping Beauty-Induced Tumors
Source: PLoS One. 2011 Sep 13;6(9):e24668. doi: 10.1371/journal.pone.0024668 (PMC3172244; doi:10.1371/journal.pone.0024668)
Supplement: Table S2 — Comparison of cutoff methods in the identification of CIS and gCIS genes. (PDF) [file pone.0024668.s009.pdf]

Supplemental Table S2. Comparison of cutoff methods in the identification of CIS and gCIS genes.

	NB			1% max			0.1% total			Dynamic		
	# of sites	CIS	%	# of sites	CIS	%	# of sites	CIS	%	# of sites	CIS	%
Vav-SB	10,543	27	33	11,544	26	31	5,271	24	42	2,657	21	43
CD4-SB	21,700	72	24	25,517	77	23	4,561	48	29	3,735	44	34

	NB			1% max			0.1% total			Dynamic		
	# of sites	gCIS	%	# of sites	gCIS	%	# of sites	gCIS	%	# of sites	gCIS	%
Vav-SB	10,543	43	30	11,544	29	28	5,271	43	26	2,657	30	37
CD4-SB	21,700	193	11	25,517	205	12	4,561	107	20	3,735	88	24

% = percentage of genes found in COSMIC and CGC databases (see Methods for definition of mutated COSMIC genes)
