# Supplementary material for: Novel Molecular and Computational Methods Improve the Accuracy of Insertion Site Analysis in Sleeping Beauty-Induced Tumors
Source: PLoS One. 2011 Sep 13;6(9):e24668. doi: 10.1371/journal.pone.0024668 (PMC3172244; doi:10.1371/journal.pone.0024668)
Supplement: Table S3 — Comparison of CIS genes identified by 454 and Illumina sequencing in SB-induced lymphomas. (PDF) [file pone.0024668.s010.pdf]

Table S3. Comparison of CIS genes identified by 454 and Illumina sequencing in SB-induced lymphomas.

Gene symbol	Vav-SB Illumina	Vav-SB 454	CD4-SB Illumina	CD4-SB 454
Ghr	•	•	•	•
Myc	•	•	•	•
Sos1	•	•	•	•
Stat5b	•	•	•	•
Akt2	•		•	•
Ets1	•	•	ns	•
Foxp1	•	•	•	
Rasgrf1	•	•	•	
Runx1	•	•	•	
Zmiz1	•	•	•	
Akt1	•		ns	•
Ambra1			•	•
Bcar3			•	•
Birc6			•	•
Brd4			•	•
Ccnd3			•	•
Cdkn2a			•	•
Crebbp	•		•	
Erg	•	•		
Flt3	•	•		
Gfi1			•	•
Hipk2			•	•
Hivep2			•	•
Ikzf1	•	•		
Jak1			•	•
Mbnl1			•	•
Ncoa2			•	•
Notch1	•	•		
Nsd1			•	•
Pan3			•	•
Phf21a			•	•
Pik3r5			•	•
Prlr	•	•		
Raf1			•	•
Rasgrp1	•	•		
Satb1			•	•
Sp3			•	•
Wac			•	•
Whsc1			•	•
Wnk1			•	•
Zmynd11	•	•		
2010007H12Rik			•	
2700078E11Rik			ns	•
4931406P16Rik			•	
5730419I09Rik			•	
Adam10			ns	•
Ankrd11			•	
Appbp2			•	
Arhgap17			ns	•
Arhgef3			•	
Arid4a			•	
Asap1			ns	•
Bmi1	•			
Cdh23	•			
Chd9			•	
Chst11			•	
Cnot2			•	
Cnot4			•	
Col5a1	•			
Csnk1g1			•	
D14Abb1e			•	
Ehd3			•	
Eif3h			ns	•
Epc2	•			
Eps8			ns	•
Exoc2			•	
Fam53b			•	
Fyn			•	
Gab2	•			
Gpatch8			•	
Gsk3b			•	
Herc1	•			
Hnrnpc			•	
Hnrnpd			•	
Il2rb	•			
Ipcef1			ns	•
Iqgap2			ns	•
Irf4			•	
Kis2			•	
Kpna4			•	
Lnpep			ns	•
Lonp2			•	
Lrrc16a	•			
Lrrc8c			ns	•
Malt1			•	
Man1a			•	
Manba			•	
Map3k12, Pcbp2			nd	•
Map3k4			•	
Map3k5			•	
Memo1			•	
Mta3			•	
Mtfr1			•	
Myo16	•			
Ncoa3			•	
Nedd9			•	
Nf1	•			
Nfkb1			•	
Nipbl			•	
Nrcam	•			
Odz2	ns	•		
Pcm1			•	
Pdia5			ns	•
Pdpk1			•	
Picalm			•	
Plcb4			ns	•
Ptpn22			•	
Rap1gds1			ns	•
Rassf3			•	
Rftn1			•	
Rtel1			ns	•
Setd2			•	
Sfi1			•	
Sfmbt2			ns	•
Sfrs15			•	
Sik3	•			
Sin3a	•			
Slc36a4			•	
Slmap	ns	•		
Smarca2			•	
Smg6			•	
Ss18			•	
Strbp			ns	•
Taf4a			•	
Tbc1d1			•	
Tbc1d22b			•	
Tlk1			•	
Tmem164			•	
Tox2	ns	•		
Ttc3			•	
Ubn2			•	
Zfand3			•	
Zfx			•	
Zswim6			•	

ns = identified by Illumina sequencing but not mutated at significant rate

nd = not identified by Illumina sequencing
