# Supplementary material for: Novel Molecular and Computational Methods Improve the Accuracy of Insertion Site Analysis in Sleeping Beauty-Induced Tumors
Source: PLoS One. 2011 Sep 13;6(9):e24668. doi: 10.1371/journal.pone.0024668 (PMC3172244; doi:10.1371/journal.pone.0024668)
Supplement: Methods S1 — Detailed Illumina-based LM-PCR protocol. (PDF) [file pone.0024668.s011.pdf]

Preparation of templates for Illumina sequencing

Restriction digest

1. Digest 1µg of tumor DNA with *Nla*III (IRR) or AluI (IRL). Do not use more than 2 µg of genomic DNA as this will lead to concatomerization of genomic fragments during the ligation step. Less than 1 µg of genomic DNA can be used, but the final volume should be scaled to maintain a similar DNA concentration in the reaction.

   1 µL enzyme
   4 µL buffer
   4 µL 10X BSA (if needed)
   X µL H_2O
   Y µL DNA
   20 µL Total

2. Incubate at least 3 hours at 37˚C.
3. Heat inactivate enzyme.

The restriction digest can be incubated overnight. In this case, heat inactivation of the enzyme is not required. However, overnight incubation should be performed in a 37˚C incubator (not a water bath) to prevent evaporation of the sample.

Ligation

1. Prepare the adaptor by mixing the linker+ and linker- primers (each at 100µM) at a 1:1 ratio (see below for primer details). Linkers should be re-suspended at 100µM when stored. Heat the primer solution at 95˚C for 5 minutes. Turn off the heat and allow the primers to slowly cool to room temperature. This allows the single-stranded oligos to anneal and form the double-stranded adaptor.

2. Set up ligations:

10.0 µL	digested genomic DNA
2.0 µL	10X NEB buffer 4
2.0 µL	10 mM ATP
1.5 µL	adaptor
1.0 µL	T4 ligase (2,000U)
3.5 µL	dH_2O
**20.0 µL**	**Total**

   ligate 2-3 hours at room temperature or overnight at 16˚C

3. Heat inactivate the T4 ligase (65˚C for 10 minutes).

4. Digest ligation with *BamHI*. This prevents the fragment from transposons within the concatomer from being amplified. *BamHI* solution is made in a 10µL volume per tube. To each tube add:

   1.0 µL *Bam*HI
   1.0 µL NEB Buffer 4
   3.0 µL 10X BSA
   5.0 µL dH_2O
   10.0 µL Total

If the digest ligation is performed overnight at 37˚C then column purification is not required as the *Bam*HI has degraded. Otherwise, a column purification is needed to remove the *Bam*HI enzyme.

PCR

2.00 µL	ligation reaction	Step 1	98˚C	30 seconds
10.00 µL	5X buffer			
1.00 µL	10 mM dNTPs	Step 2	98˚C	10 seconds
1.50 µL	primer 1 (10 µM)		63˚C	20 seconds
1.50 µL	primer 2 (10 µM)		72˚C	30 seconds
0.25 µL	Phusion polymerase (NEB)		repeat Step 2 for 25 cycles	
33.75 µL	H2O			
50.00 µL	Total	Step 3	72˚C	2 minutes
			Hold at 4˚C	

- dilute 3 µL of PCR reaction in 147 µL H_2O (1:50 dilution)
- store remaining primary PCR reaction at 4˚C

Set up secondary PCR

4.00 µL	diluted primary PCR (diluted 1:50 in H_2O)
20.00 µL	5X buffer
2.00 µL	10 mM dNTPs
3.00 µL	nested primer 1 (10 µM)
3.00 µL	nested primer 2 (10 µM)
1.00 µL	Phusion polymerase (NEB)
67.00 µL	H2O
100.00 µL	Total

- perform PCR using the same cycle conditions as primary PCR (25 cycles)

- Analyze 25 µL of PCR product on 1.5% agarose gel.
- Purify remaining PCR product to remove excess primers/dNTPs.
- Determine concentration of purified PCR products (Nanodrop or UV spec is sufficient)
- Pipet 25 ng of each PCR product pool into a single tube to be run on a single lane on the Illumina platform
- Adjust the final concentration of the mixed sample to be ~20-25 ng/µL.
- Incubate the diluted products at 37-42˚C for 30 minutes
- Submit sample for sequencing

Primers to generate adaptors:
<u>IRDRR adaptor</u>
NlaIII linker+ 5'-GTAATACGACTCACTATAGGGCTCCGCTTAAGGGACCATG-3'
NlaIII linker- 5'-Phos-GTCCCTTAAGCGGAG-C3spacer-3'

<u>IRDRL adaptor</u>
AluI linker+ 5'-GTAATACGACTCACTATAGGGCTCCGCTTAAGGGAC-3'
AluI linker- 5'-Phos-GTCCCTTAAGCGGAG-C3spacer-3'

All adaptor primers are resuspended in STE* buffer at 100µM. All PCR primers were used at 10 µM concentration. C3spacer modification is available from Integrated DNA Technologies.

Primers for IRR amplification (NlaIII-digested DNA):

<u>Primary PCR</u>
IRR 5'GGATTAAATGTCAGGAATTGTGAAAA 3'
linker primer 5'GTAATACGACTCACTATAGGGC 3'

Primers for IRL amplification (BfaI and AluI-digested):
<u>Primary PCR</u>
IRL 5'AAATTTGTGGAGTAGTTGAAAAACGA 3'
linker primer 5'GTAATACGACTCACTATAGGGC 3'

<u>Secondary PCR (for IRR and IRL)</u>
IR-A1
5'AATGATACGGCGACCACCGAGATCTACACTCTTTCCCTACACGACGCTCTTCCGATCT(barcode)
TGTATGTAAACTTCCGACTTCAACTG

Linker-A2
5'CAAGCAGAAGACGGCATACGAGCTCTTCCGATCTAGGGCTCCGCTTAAGGGAC 3'

see info on barcoded primers

<u>*STE Buffer</u>
50 mM NaCl
10 mM Tris-Cl (pH 8.0)
1mM EDTA (pH 8.0)
